# On the Composition of Human Milk in Health and Disease

**Published:** 1853-06

**Authors:** A. Becquerel


*On the Composition of Human Milk in Health and Disease.* By MM. Vernois and A. Becquerel.—Looking at the contradictory reports of various analyses of milk, MM. Vernois and A. Becquerel have entered into an elaborate investigation of the entire subject. They have especially chosen 89 uniform and complete analyses to deduce certain deductions from. The following is their account of the composition of this fluid:

| | In Health. | | In Acute Disease. | | In Chronic Disease. |
|---|---|---|---|---|---|
| Water.............. | 889 08 | .. | 884·91 | .. | 885-50 |
| Solid parts......... | 110·92 | .. | 115·09 | .. | 114·50 |
| Sugar............... | 43 64 | .. | 33·10 | .. | 43 37 |
| Caseum & extractive | 39·24 | .. | 50·40 | .. | 37-66 |
| Butter.............. | 26-66 | .. | 29-86 | .. | 32 57 |
| Salts (by incineration) | 1-38 | .. | 1-73 | .. | 1·50 |
| Density............ | 1032-67 | .. | 1031-20 | .. | 1031-47 |

There are more solid parts in the milk of nurses aged from 15 to 20, than in those of from 35 to 40. The quantity of butter is notably increased during the colostral period. Gestation does not induce alteration in the composition of the milk at first, but at a later period it increases the proportion of solid parts. Menstruation diminishes the density, the weight of the water and of the sugar. It increases the weight of the solid portions, especially the caseum. Insufficient aliment renders the milk too watery, the effect falling especially on the butter and caseum. An excess of butter or caseum always accompanies an ill state of health of the nursling. There are certain women whose milk, independently of any special cause, always contains an excess of butter or caseum.

In both acute and chronic disease the water diminishes and the solid parts increase; but there the analogy between these two classes ceases. In acute disease, the sugar considerably diminishes, while the three other elements are increased, the caseum alone nearly repairing what is lost by the sugar. In chronic disease, the butter and salts are increased, the sugar remains sta'ionary, and the caseum diminishes. Thus, in acute diseases, we have loss on a respiratory element, and excess in a nutritive element; and in the chronic, loss on the nutritive element, and increase of the respiratory element. In phthisis, without diarrhœa or emaciation, there is little sensible modifieation; but these being present, there is considerable diminution in the weight of butter. In syphilis, the density is extraordinarily raised; the butter diminishes, and the salts disproportionately increase.—*Gazette Médicale*, 1853, No. 5.

*On the Influence of Posture in the Treatment of Epilepsy.* By Dr. Marshall Hall.—We have only to raise one hand and arm high above the head, and allow the other to hang down, for a minute or two, and then bring the hands together and compare the syncopal condition of the former with the apoplectic condition of the latter, to form an idea of the influence of posture in the treatment of diseases consisting of affections of the circulation, especially that of the head.

I believe ordinary syncope may pass into fatal sinking if the raised posture be continued.

I believe that simple apoplexy may become deeper and deeper, simply from the opposite course of retaining the patient in the recumbent position.

Sleep, which is a sub-apoplexy, may pass into epilepsy or apoplexy, solely from the fact of a recumbent position. As a preventive of epilepsy and apoplexy during sleep, it is of the utmost moment that the patient should habitually repose with the head and shoulders much raised. For this purpose both bed and mattress should be raised by means of a bed-chair, or triangular cushion, and the patient be prevented from gliding down in the bed by means of a firm bolster, four inches in diameter, placed under the sheet, under the front of the ischia. The trunk should be raised to an angle of 45 or 50 degrees.

In this manner the encephalon will be less oppressed with blood, the sleep will be lighter, the disposition to epilepsy or apoplexy will be diminished.

This should be the patient's habit during the rest of life.

There are two other circumstances in which attention to posture is most important.

The *first* is the condition of the patient after certain fits of epilepsy, the respiration being impeded by *rattles* in the throat. The posture should be much raised; but, besides this, it should not be such that the saliva may *fall* into the fauces. The stupor and insensibility prevent the patient from swallowing. The saliva, therefore, if a just position be not adopted, accumulates and falls into the fauces, and a throat-rattle and dyspnœa, painful to witness, and dangerous to life, are the consequence. The posture of the patient should be such as to allow the saliva to flow out of the corner of the mouth. In one case such a change of posture relieved the patient immediately.

The *second* case requiring extreme attention to the posture of the patient is that of *Syncopal Epilepsy,* or that form of epilepsy in which there is ghastly pallor of the countenance and other signs of syncopal affection. The patient should be placed with the head *low.* If this be not done, the syncope may be speedily fatal, an event which actually occurred in an interesting case a few days only ago.

The patient was no other than Ann Ross, on whom Mr. Anderson had performed the operation of tracheotomy. Her fits had changed from those of the epilepsia *laryngea* to the abortive form. The reader may remember that the patient's age was thirty-six ; that her case was hereditary, her father having been epileptic ; and inveterate, her fits having recurred during twenty-four years; and that she herself was thin and pallid. She was seized with syncopal epilepsy ; was laid on the bed and expected to recover as formerly ; was left ; and was at length found to have expired. A low position and proper attention might have saved the poor creature's life.

I need scarcely observe, that what I have said of epilepsy applies to many other diseases. It is the *principle* of position which I wish to enforce; a principle, the importance of which I believe to be still greater and still more extensive in application than is generally imagined. *London Lancet.*

